# Supplementary material for: Atmospheric oxidation drove climate change on Noachian Mars
Source: Nat Commun. 2024 Jul 5;15:5648. doi: 10.1038/s41467-024-47326-0 (PMC11226428; doi:10.1038/s41467-024-47326-0)
Supplement: Supplementary file 1 — Supplementary Information file [file 41467_2024_47326_MOESM1_ESM.pdf]

Supplemental Information

Atmospheric oxidation drove climate change on Noachian Mars

Jiacheng Liu*[1,2], Joseph R. Michalski*[1], Zhicheng Wang[1], Wen-Sheng Gao[3]

[1]Department of Earth Sciences and Laboratory for Space Research, The University of Hong Kong, Hong Kong, China

[2]NWU-HKU Joint Center of Earth and Planetary Sciences, Department of Earth Sciences, The University of Hong Kong, Hong Kong, China

[3]School of Earth Resources, China University of Geosciences, Wuhan 430074, China

Correspondence author: Joseph Michalski, jmichal@hku.hk and Jiacheng Liu, jcliu01@hku.hk

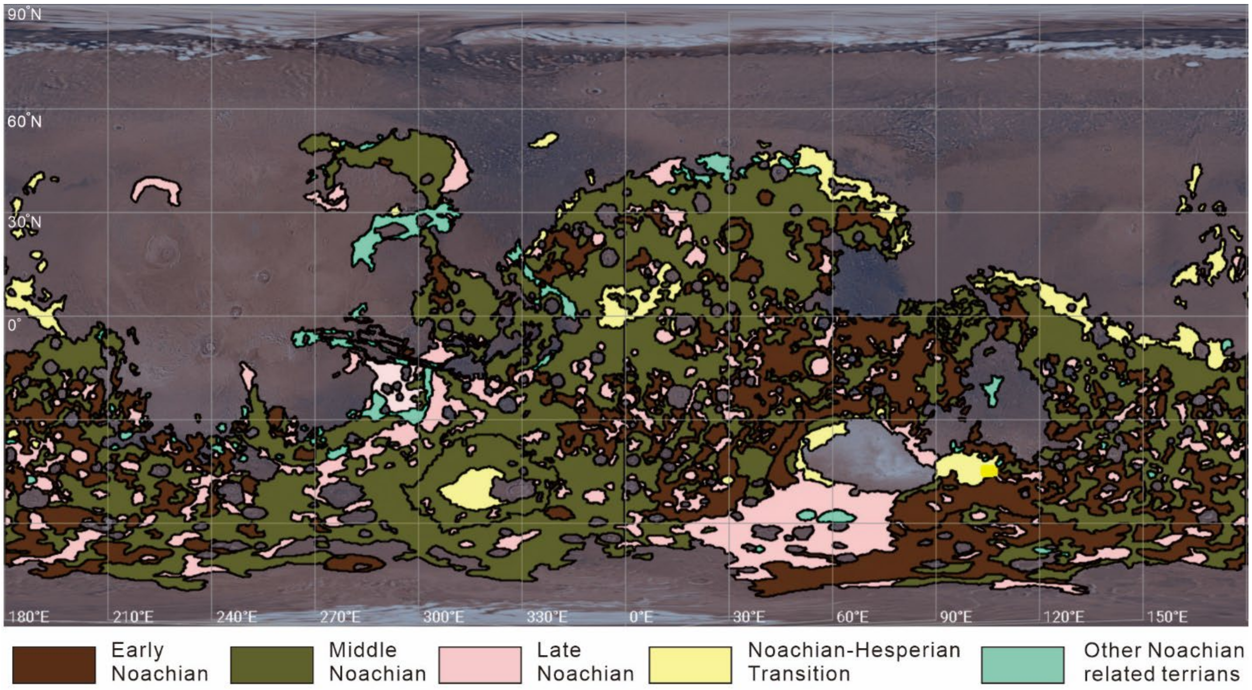

Supplementary Figure 1. Global Noachian units in a Mars global map produced by Tanaka et al. (2014)[38](https://pubs.usgs.gov/sim/3292/) over the Viking colorized global mosaic 232 m v2 (NASA/JPL/USGS/AMES,

https://astrogeology.usgs.gov/search/map/Mars/Viking/MDIM21/Mars_Viking_MDIM21_ClrM

osaic_global_232m). Early Noachian includes Early Noachian highland unit, Early Noachian highland massif unit. Middle Noachian includes Middle Noachian highland unit and Middle Noachian highland massif unit. Late Noachian includes late Noachian volcanic unit. Hesperian and Noachian transition include Hesperian and Noachian transition unit, Hesperian and Noachian highland undivided unit, and Hesperian and Noachian basin unit. Other Noachian related terrains include Noachian volcanic edifice unit, Noachian highland undivided unit, Noachian highland edifice unit, and Amazonian and Noachian apron unit.

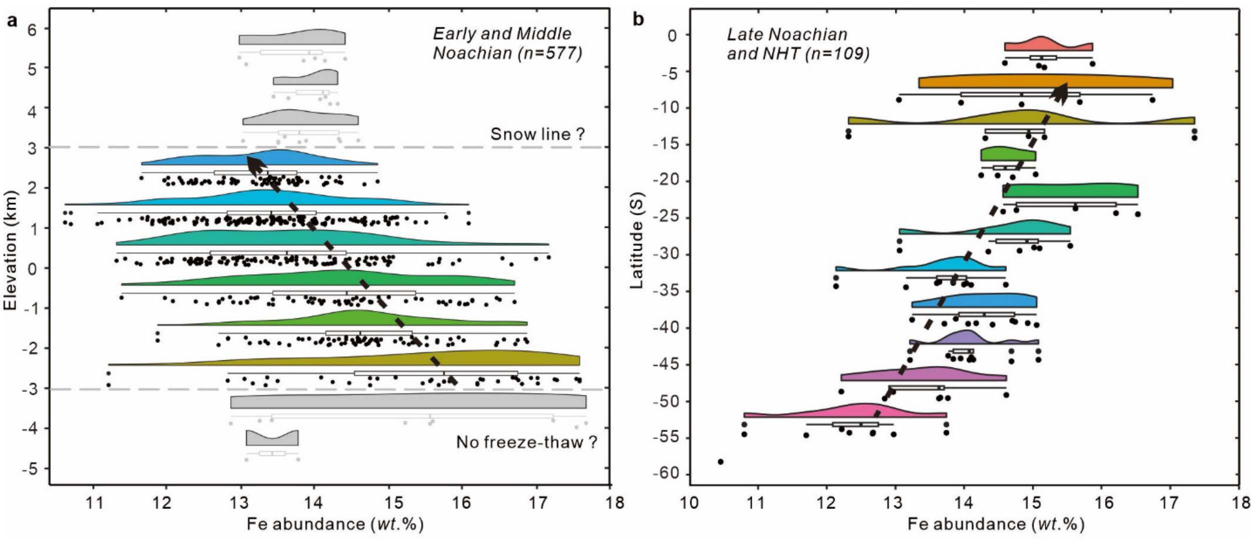

Supplementary Figure 2. Raincloud plot of surface Fe abundances across different elevations in the Early and Middle Noachian terrains (a) and across different latitude (b) in the Late Noachian and Noachian-Hesperian Transition (NHT) terrains. Source data are provided as a Source Data file.

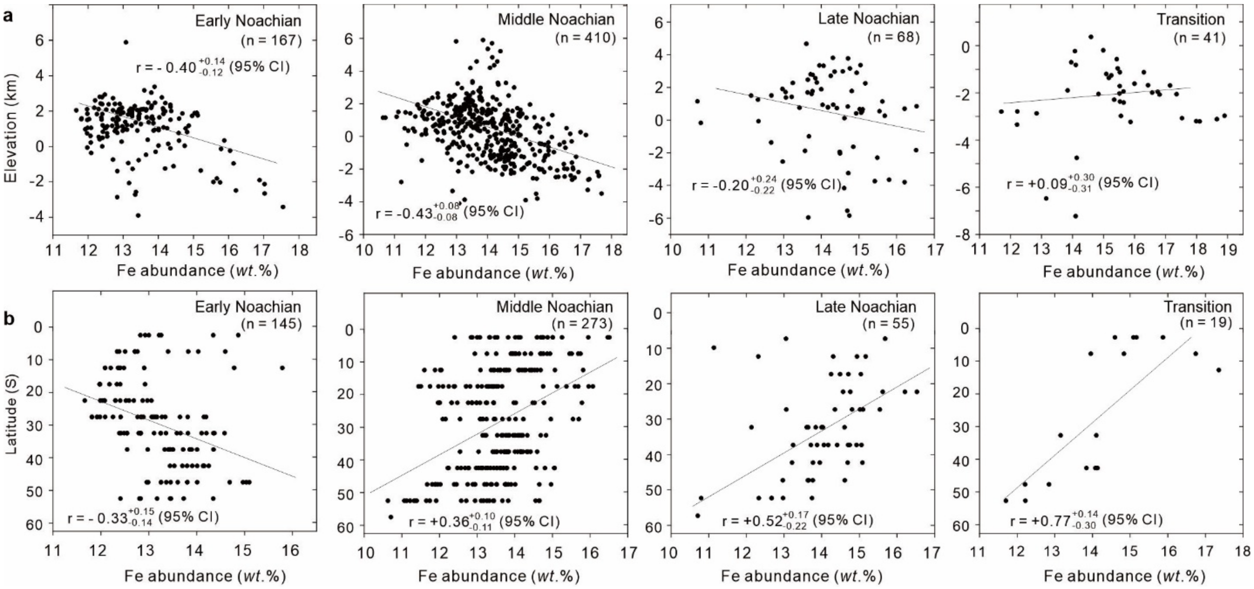

Supplementary Figure 3. The scattered graphs between Fe abundance, elevation (a), and latitude (b) at the surface of terrains of different epochs on Mars. The correlation coefficient (r) between Fe abundance and latitude is analyzed mainly for the southern hemisphere because there is significant elevation variation in the northern hemisphere. Source data are provided as a Source Data file.

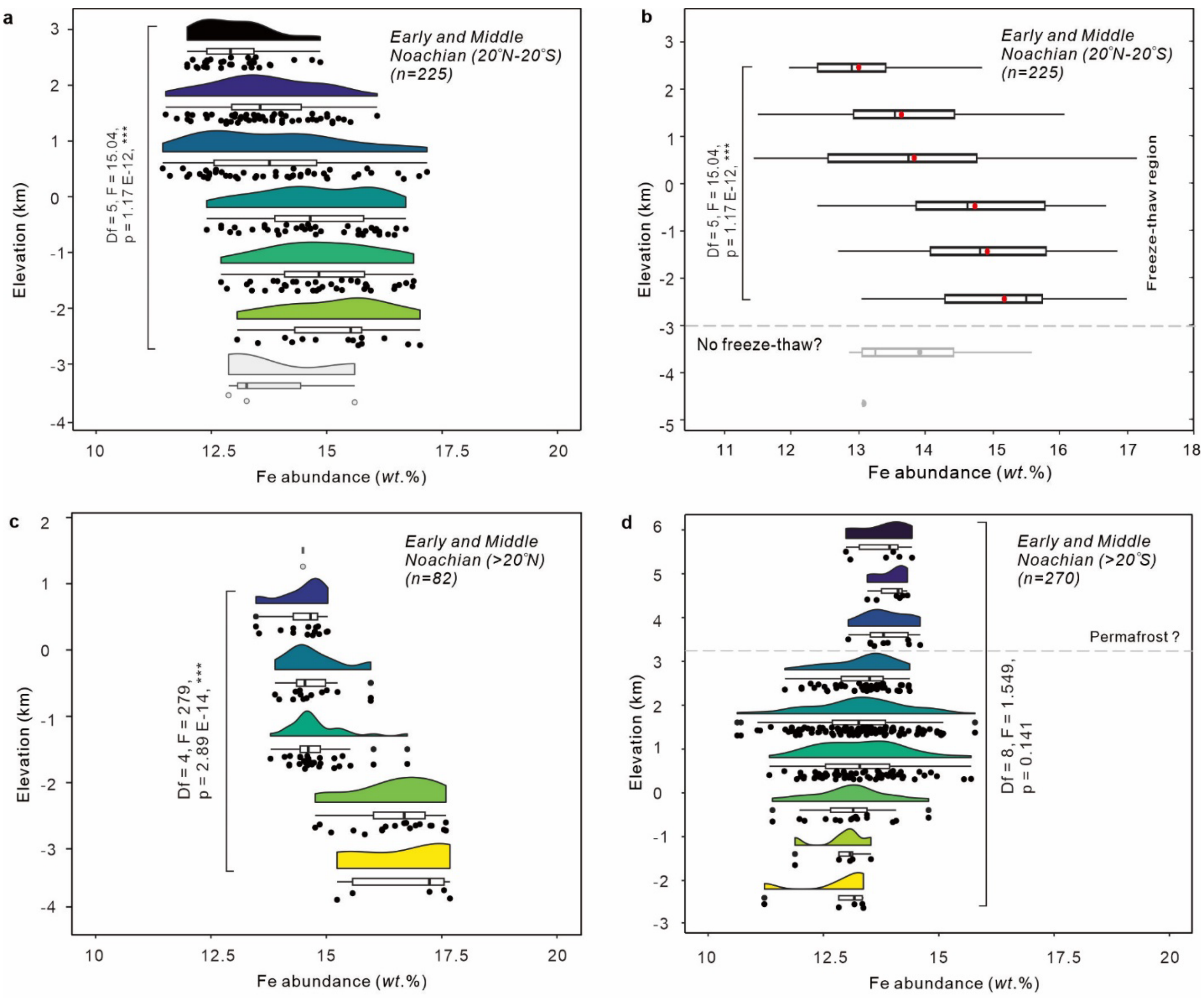

Supplementary Figure 4. Raincloud plot of surface Fe abundances across different elevation in different latitude bands in the Early and Middle Noachian terrains. Source data are provided as a Source Data file.

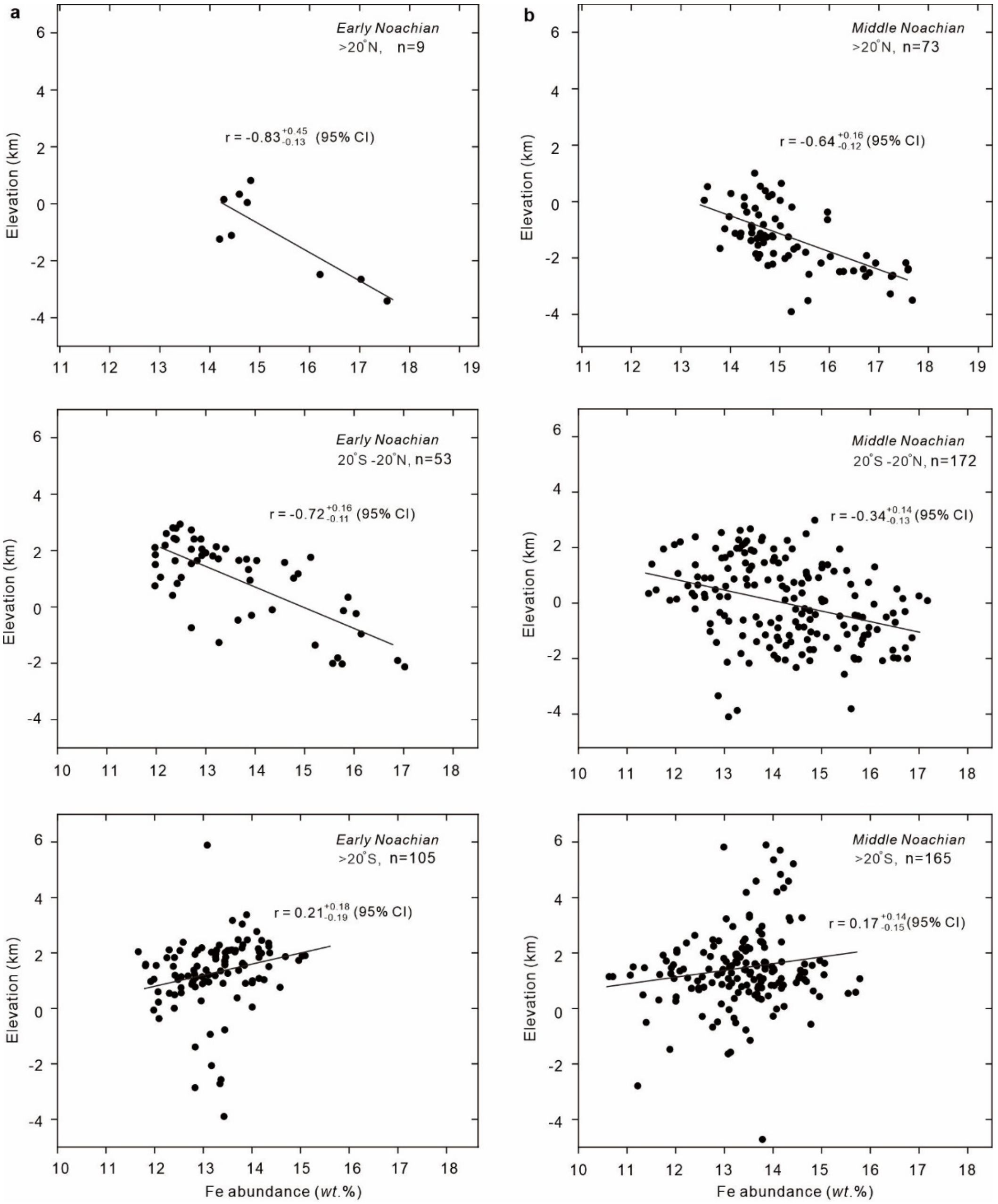

Supplementary Figure 5. The scattered graphs between surface Fe and elevation of Early (a) and Middle (b) Noachian terrains on Mars in three latitudinal bands (>20°N, 20°S-20°N, >20°S). Source data are provided as a Source Data file.

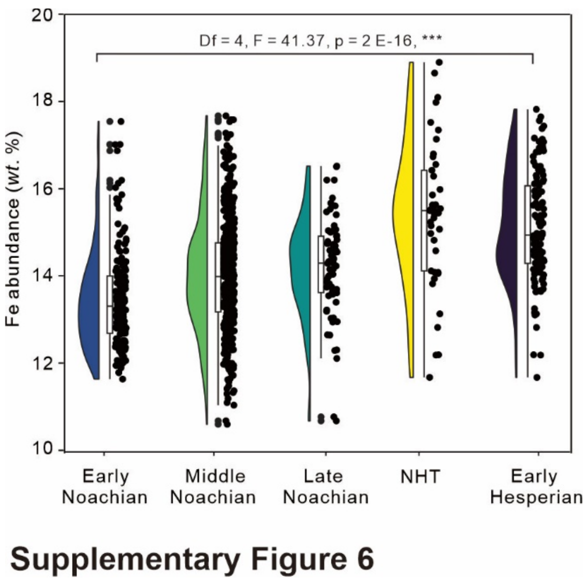

Supplementary Figure S6. **Raincloud plot of Fe abundances across different age categories from Early Noachian to Early Hesperian.** Source data are provided as a Source Data file.

Supplementary Table 1. ANOVA results on Fe abundances between different elevation categories in the Early and Middle Noachian terrains.

-	Df	Sum Sq	Mean Sq	F value	Pr (>F)	Signif. codes
Elevation categories	5	244.8	48.96	39.02	2E-16	***
Residuals	539	676.4	1.25			

Codes: 0 '***' 0.001 '**' 0.01 '*' 0.05 '.' 0.1 ' ' 1

Note: Elevation groups (3000 to 6000 and -5000 to -3000) were excluded from the ANOVA analysis.

Supplementary Table 2. ANOVA results on Fe abundances between different elevation categories in the Early and Middle Noachian terrains (>20°N).

-	Df	Sum Sq	Mean Sq	F value	Pr (>F)	Signif. codes
Elevation categories	4	56.57	14.142	279	2.89E-14	***
Residuals	76	38.52	0.507			

Codes: 0 '***' 0.001 '**' 0.01 '*' 0.05 '.' 0.1 ' ' 1

Note: Elevation groups (1000 to 2000 m) were excluded from the ANOVA analysis due to their limited number of data points.

Supplementary Table 3. ANOVA results on Fe abundances between different elevation categories in the Early and Middle Noachian terrains (20°N-20°S).

-	Df	Sum Sq	Mean Sq	F value	Pr (>F)	Signif. codes
Elevation categories	5	107.4	21.485	15.04	1.17E-12	***
Residuals	215	30732	1.429			

Codes: 0 '***' 0.001 '**' 0.01 '*' 0.05 '.' 0.1 ' ' 1

Note: Elevation groups (-3000 to -4000 and -4000 to -5000) were excluded from the ANOVA analysis due to their limited number of data points .

Supplementary Table 4. ANOVA results on Fe abundances between different elevation categories in the Early and Middle Noachian terrains (>20°S).

-	Df	Sum Sq	Mean Sq	F value	Pr (>F)	Signif. codes
Elevation categories	8	9.89	1.2357	1.55	0.141	
Residuals	259	206.6	0.7977			

Codes: 0 '***' 0.001 '**' 0.01 '*' 0.05 '.' 0.1 ' ' 1

Note: Elevation groups (-3000 to -4000) and (-4000 to -5000) were excluded from the ANOVA analysis due to their limited number of data points (< 3).

Supplementary Table 5. ANOVA results on Fe abundances between different latitude categories in the Late Noachian and NHT terrains.

-	Df	Sum Sq	Mean Sq	F value	Pr (>F)	Signif. codes
Latitude categories	10	52.11	5.211	6.67	6.74E-07	***
Residuals	61	47.65	0.781			

Note: Latitude groups (-55°S to -60°S) were excluded from the ANOVA analysis due to their limited number of data points (< 3).

Supplementary Table 6. ANOVA results on Fe abundances between different age categories

	Df	Sum Sq	Mean Sq	F value	Pr (>F)	Signif. codes
Age	4	272.4	68.1	41.37	<2E-16	***
Residuals	810	1333.4	1.65			

Supplementary Table 7. Z-test results on Fe abundances between different age categories

Pairs	Z_value	p_value	Signif. codes
Early Hesperian vs NH Transition	-0.89	0.3720	
Early Hesperian vs Late Noachian	5.33	0.0000	***
Early Hesperian vs Middle Noachian	8.67	0.0000	***
Early Hesperian vs Early Noachian	12.01	0.0000	***
NH Transition vs Late Noachian	4.02	0.0001	***
NH Transition vs Middle Noachian	4.97	0.0000	***
NH Transition vs Early Noachian	6.94	0.0000	***

Late Noachian vs Middle Noachian	0.97	0.3338	
Late Noachian vs Early Noachian	4.38	0.0000	***
Middle Noachian vs Early Noachian	5.26	0.0000	***
